# Supplementary material for: A defined bacterial consortium and spatial transcriptomics highlight the complex interaction between Campylobacter jejuni and the murine intestine
Source: Gut Microbes. 2025 Dec 18;17(1):2600053. doi: 10.1080/19490976.2025.2600053 (PMC12931722; doi:10.1080/19490976.2025.2600053)
Supplement: Supplementary Material — Supplementary Figure 1. Pathway analysis of Clusters 2,3, and 17 differentially expressed genes in the host. (A) Upregulated pathways in Cluster 2, Downregulated pathways in Cluster 2, (B) Upregulated pathways in Cluster 3, Downregulated pathways in Cluster 3, (C) Upregulated pathways in Cluster 17, Downregulated pathways in Cluster 17. Qiagen IPA software was used to identify significantly altered pathways (Padj value < 0.05).Supplementary Figure 2. C. jejuni infection drives spatial enrichment of myeloid cells in the colon (A–C) Spatial cell-type deconvolution of major cell types in colonic Swiss roll sections from (A) germ-free (GF), (B) C13-colonized, and (C) C13 + C. jejuni-infected mice. [file KGMI_A_2600053_SM6554.docx]

**Supplementary Figures**

**Supplementary Figure 1**. Pathway analysis of Clusters 2,3, and 17 differentially expressed genes in the host. (A) Upregulated pathways in Cluster 2, Downregulated pathways in Cluster 2, (B) Upregulated pathways in Cluster 3, Downregulated pathways in Cluster 3, (C) Upregulated pathways in Cluster 17, Downregulated pathways in Cluster 17. Qiagen IPA software was used to identify significantly altered pathways (*P*_adj_ value < 0.05).

**Supplementary Figure 2**. *C. jejuni* infection drives spatial enrichment of myeloid cells in the colon (A–C) Spatial cell-type deconvolution of major cell types in colonic Swiss roll sections from (A) germ-free (GF), (B) C13-colonized, and (C) C13 + *C. jejuni*-infected mice.
